# A regularity index for dendrites - local statistics of a neuron's input space

**DOI:** 10.1371/journal.pcbi.1006593

**Published:** 2018-11-12

**Authors:** Laura Anton-Sanchez, Felix Effenberger, Concha Bielza, Pedro Larrañaga, Hermann Cuntz

**Affiliations:** 1 Departamento de Inteligencia Artificial, Universidad Politécnica de Madrid, Madrid, Spain; 2 Ernst Strüngmann Institute (ESI) for Neuroscience in Cooperation with Max Planck Society, Frankfurt/Main, Germany; 3 Frankfurt Institute for Advanced Studies (FIAS), Frankfurt/Main, Germany; Research Center Jülich, GERMANY

## Abstract

Neurons collect their inputs from other neurons by sending out arborized dendritic structures. However, the relationship between the shape of dendrites and the precise organization of synaptic inputs in the neural tissue remains unclear. Inputs could be distributed in tight clusters, entirely randomly or else in a regular grid-like manner. Here, we analyze dendritic branching structures using a regularity index *R*, based on average nearest neighbor distances between branch and termination points, characterizing their spatial distribution. We find that the distributions of these points depend strongly on cell types, indicating possible fundamental differences in synaptic input organization. Moreover, *R* is independent of cell size and we find that it is only weakly correlated with other branching statistics, suggesting that it might reflect features of dendritic morphology that are not captured by commonly studied branching statistics. We then use morphological models based on optimal wiring principles to study the relation between input distributions and dendritic branching structures. Using our models, we find that branch point distributions correlate more closely with the input distributions while termination points in dendrites are generally spread out more randomly with a close to uniform distribution. We validate these model predictions with connectome data. Finally, we find that in spatial input distributions with increasing regularity, characteristic scaling relationships between branching features are altered significantly. In summary, we conclude that local statistics of input distributions and dendrite morphology depend on each other leading to potentially cell type specific branching features.

## Author summary

Dendritic tree structures of nerve cells are built to optimally collect inputs from other cells in the circuit. By looking at how regularly the branch and termination points of dendrites are distributed, we find characteristic differences between cell types that correlate little with other traditional branching statistics and affect their scaling properties. Using computational models based on optimal wiring principles, we then show that termination points of dendrites generally spread more randomly than the inputs that they receive while branch points follow more closely the underlying input organization. Existing connectome data validate these predictions indicating the importance of our findings for large scale neural circuit analysis.

## Introduction

The primary function of dendritic trees is to collect inputs from other neurons in the nervous tissue [1,2]. Different cell types play distinct roles in wiring up the brain and are typically visually identifiable by the particular shape of their dendrites [3]. However, so far no branching statistic exists that reliably associates individual morphologies to their specific cell class [4,5], indicating that we have not yet identified the morphological features that are characteristic for the differences in how neurons connect to one another. Theoretical considerations have provided systematic qualitative insight into the question of how dendrite shape relates to specific connectivity. Dendrites are thought to collect their inputs using the shortest amount of cable and minimizing conduction times in the circuit [3,6–9] and they have been proposed to maximize the possible connection repertoire [10]. Of the possible connections that a neuron could make by anatomical proximity only a small, relatively invariable number become functional synapses [11]. But it has generally been proposed that the connection probability between a dendrite and an axon can be determined by the amount of anatomical overlap between the two [12–14]. Furthermore, dendrite shape has been linked to the number of synapses based on the optimal wiring assumptions described above, linking total dendrite length and the number of synapses that determine the morphology [15]. This leads to the question whether specific axonal arrangements or synapse distribution patterns may coincide with specific typical dendritic morphological characteristics [16].

A useful concept to relate dendritic trees with their underlying connectivity comes from extended minimum spanning trees (MSTs) that connect a set of target points while minimizing total cable length and path lengths in the tree toward the root where signals get integrated [6,7]. Such MSTs were shown to produce accurate dendritic morphologies when the corresponding target points were selected adequately [7,15,17,18]. This approach has previously linked the distribution of target points to actual synapse locations, as well as to the number of branch points (BPs) and termination points (TPs) [15]. Here, we study the relationship between local statistics of spatial input distributions and the respective dendritic morphology using MSTs generated on different target point distributions. In order to do this we use a regularity index *R* that measures the degree of clustering of points in a given volume based on the average nearest neighbor (NN) distance. Specifically, the statistic *R* is defined as the ratio between the observed mean NN distance of a set of points in a given volume and the expected mean NN distance of the same number of points distributed uniformly in the same volume, i.e. in a setting of complete spatial randomness. Randomly distributed points from a uniform distribution (i.e. samples from a Poisson process) thereby yield a value of *R* = 1 in the limit. When *R* > 1, NN distances are on average larger than in a uniform random distribution, meaning that the points are distributed more regularly. When *R* < 1, NN distances are on average shorter, indicating that the points are more clustered than expected by chance. The measure *R* has been used in a wide variety of scientific disciplines, such as physics, biology, geography and astronomy [19–22]. In neuroscience, a variant of *R* was used to describe the regular spacing of ganglion cells in the retina [23–25]. In particular, Cook performed a comparison of measures based on NN distances to study retinal mosaics [26], including *R*, which they refer to as the *dispersion index*. The statistic *R* has also been applied to graph theoretical problems such as minimum spanning trees [27], but to the best of our knowledge it has not yet been considered to characterize neuronal morphology. In the following, we first estimate *R* for BPs and TPs in real dendrites and then use MST-based morphological models to compare distributions of BPs and TPs with the underlying distribution of target points as a proxy for their corresponding synaptic input distributions.

## Results

### *R* values in branch points (BPs) and termination points (TPs) of real dendrites

**Fig 1:**
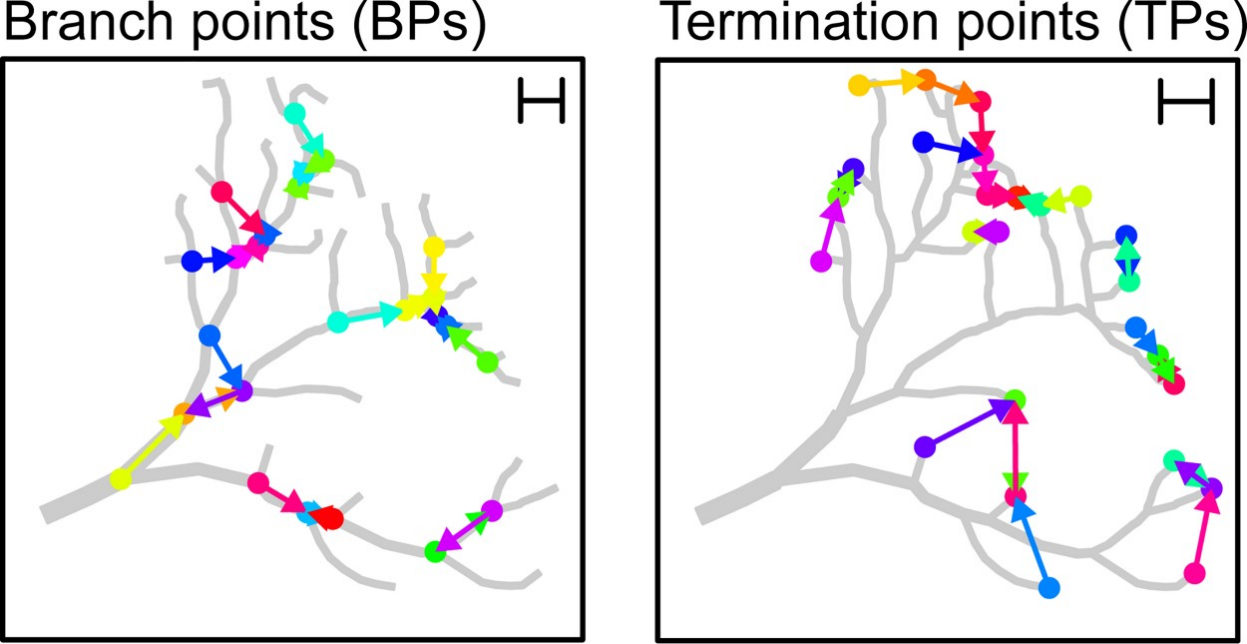
Sketch describing NN distances in BPs and TPs. Sample dendrite and NNs (colored arrows) between BPs (left) and TPs (right). The scale bar in the upper right corner shows the average NN distance, a measure that is crucial to determine the regularity index *R*.

**Fig 2:**
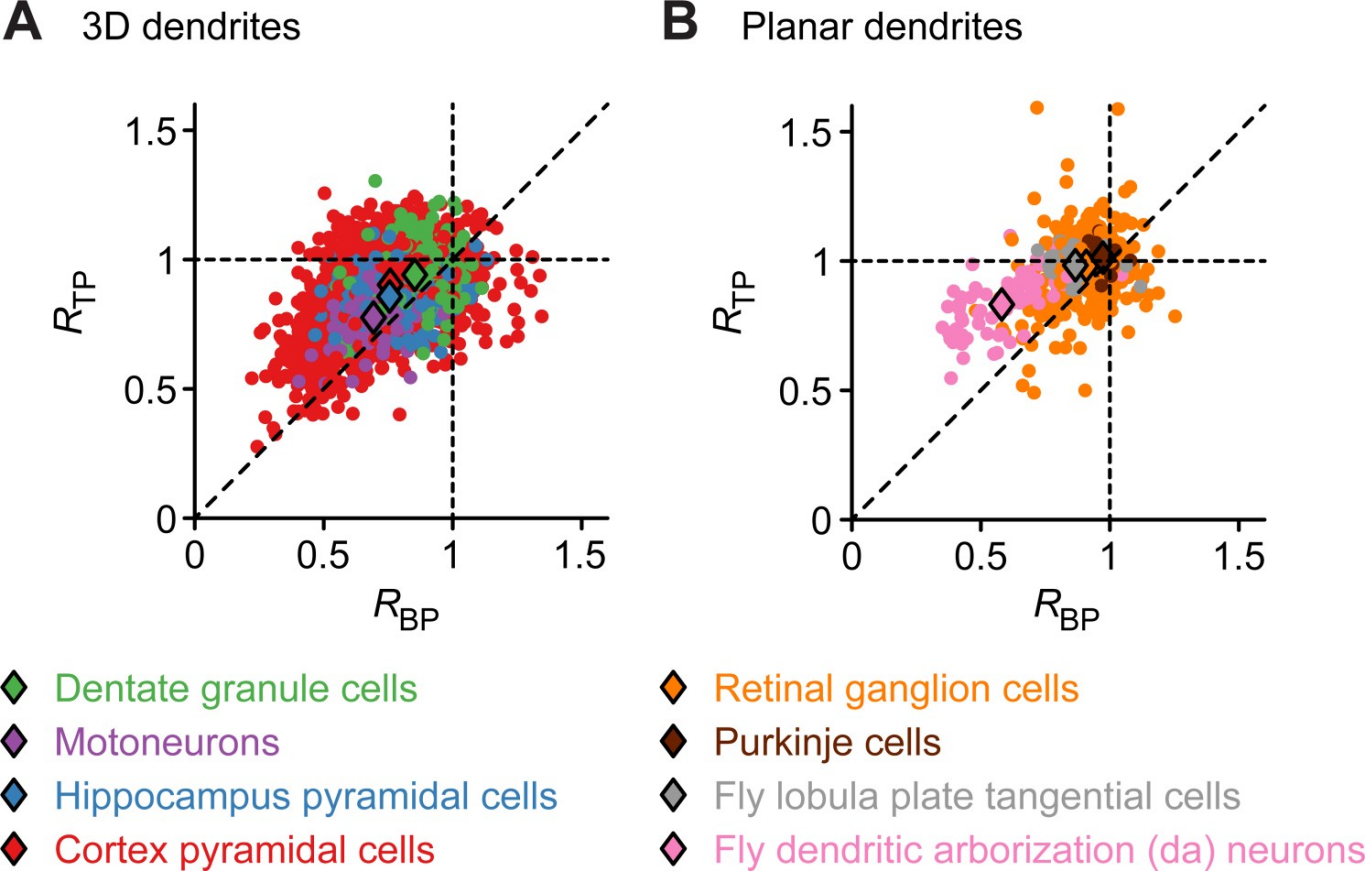
NN distances in 3D and 2D dendrites of real neurons. **A.** *R* values of BPs (x-axis) and TPs (y-axis) for four different mixed populations of 3D dendrites. **B.** Similar analysis as in A for four mixed populations of planar dendrites. Data were obtained from NeuroMorpho.Org [40]. Colored dots correspond to individual reconstructions and diamonds represent mean values for each cell type.

The value of the statistic *R*, defined as the quotient of the average nearest neighbor (NN) distance to the one expected from a matching uniform distribution can be obtained for any given set of points in Euclidean space. We first calculated *R* for the set of BPs and TPs in real dendrites to estimate how regularly the dendrites spread in the circuit (**Fig 1**). One issue when computing *R* on finite point clouds that has been given little attention in the literature so far is that a naïve calculation yields a biased result due to boundary effects. This bias is due to the fact that the enclosing volume of the point cloud is usually not known. In the special case of planar convex volumes a bias correction can be performed analytically [28], but these methods are not available for higher-dimensional and non-convex carrier volumes such as the ones occurring in our case. We addressed this issue by developing a Monte Carlo (MC) based approach to estimate for a given volume the reference value for a Poisson process numerically and obtain the value of *R* without edge effects. This method also provided us with specific confidence intervals for our estimates (see Methods and **S1–S4 Figs**). We then estimated *R* for BPs and TPs using our MC based approach, resulting in separate statistics *R*_*BP*_ and *R*_*TP*_ for the sets of branch and termination points, respectively (**Fig 2**).

**Fig 3:**
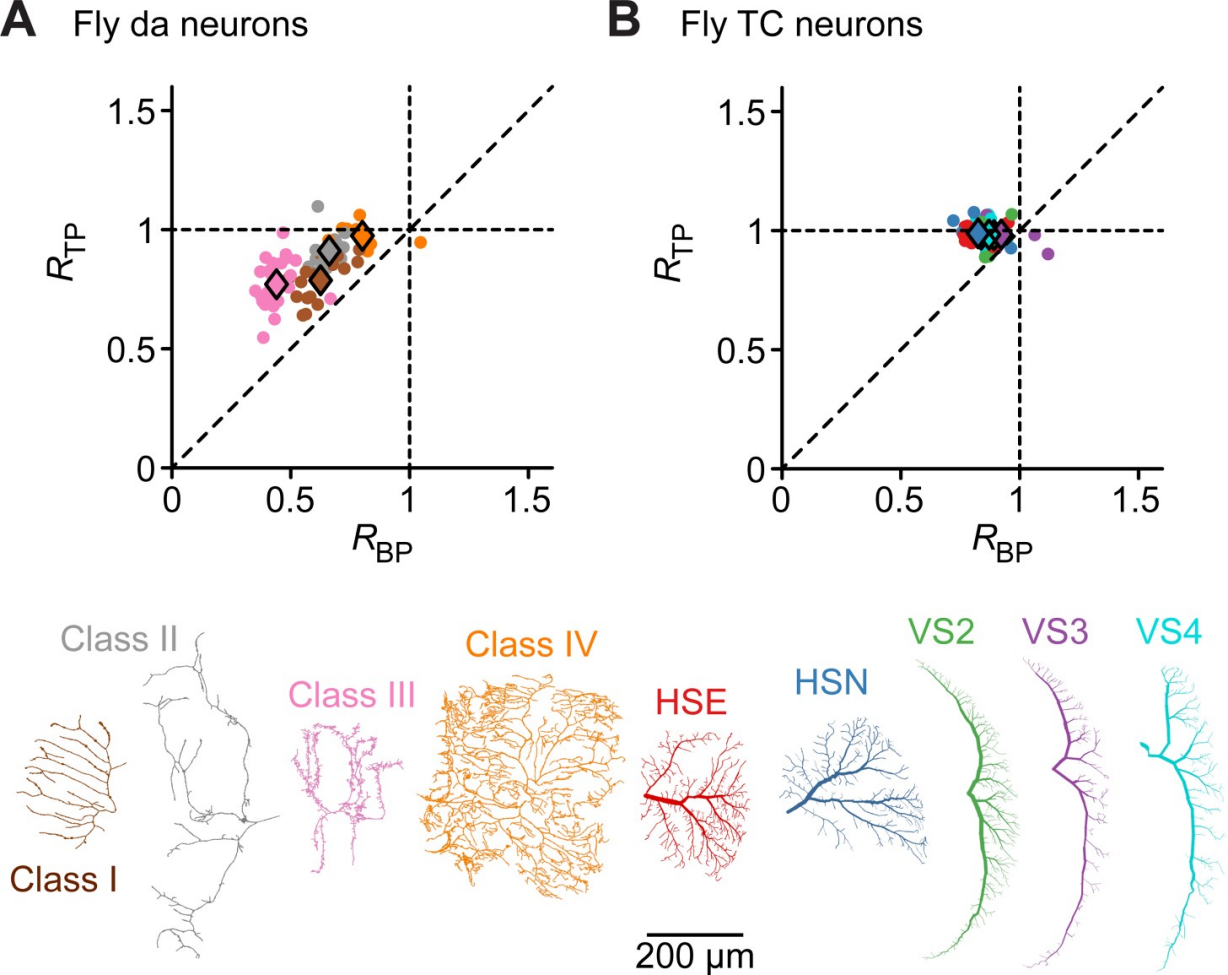
*R* values of fly da neurons and TCs subdivided into individual classes. **A.** Similar plots as in **Fig 2** but subdividing da neurons into their four different classes (I-IV). **B.** Similar subdivision as in A but for fly TCs. Here the five different cell types exhibit similar *R* values. Individual morphologies are shown to visualize the differences in how regularly the branches are distributed in the different classes.

The mean estimated values of *R* in four three-dimensional (3D) and four two-dimensional (2D) cell types varied widely. For almost all cases we observed a tendency of *R*_*TP*_ being slightly larger than *R*_*BP*_. To study whether there were significant differences between the *R*_*BP*_ and *R*_*TP*_ values of different cell types, and because not all of the necessary assumptions for ANOVA were satisfied, we used the Kruskal-Wallis test and then applied the Mann-Whitney test with the Bonferroni method to adjust the p-values for pair-wise comparisons. Using the Kruskal-Wallis test we found significant differences (p-value < 2.2 × 10^−16^) in the four tests performed, namely between the *R*_*BP*_ values in 3D cells, *R*_*TP*_ in 3D cells and the two analogous cases in 2D. In 3D dendrites, the spatial distribution of BPs and TPs was most clustered in motoneurons, followed by hippocampal pyramidal cells, neocortical pyramidal cells, and finally dentate granule cells (**Fig 2A**). Pair-wise comparisons revealed that there was no significant difference between the *R*_*BP*_ values of neocortical pyramidal cells and hippocampal pyramidal cells or between *R*_*TP*_ values of neocortical pyramidal cells and granule cells. In the case of the four planar cell types (**Fig 2B**), dendritic arborization (da) neurons in the fly larva were well characterized by the clustering of their BPs. We found no significant difference between the *R*_*TP*_ values of Lobula Plate tangential cells (TCs) in the fly, cerebellar Purkinje cells and Retinal ganglion cells, a large inhomogeneous group of cell types. Pair-wise comparison also showed no significant difference between the *R*_*BP*_ values of Purkinje cells and Retinal ganglion cells. In view of the above results, the only cell types where no differences were detected either in the *R* values for their branch or termination points were Purkinje cells and Retinal ganglion cells. However, it should be noted that our data set only contained 15 reconstructions of Purkinje cells. The results for Purkinje cells could therefore be dependent on the small number of data in that group. It is important to note that all eight populations in **Fig 2** were composed of subgroups with strong differences in their functional role in the nervous system. Moreover, morphologies within the separate subgroups were partly obtained in different species, preparations and developmental ages. To illustrate the effect this can have on the analysis, we dissected fly da neurons and TCs into their respective characteristic subgroups (**Fig 3**). Da neurons are known to subdivide into morphologically distinct classes (I–IV) and using our statistic *R* these can be separated into clusters (**Fig 3A**), corresponding to their specific *R* values. In particular, class III da neurons with their large number of small terminal segments (STSs) exhibited small *R* values consistent with the clustering of BPs and TPs due to these STSs. On the other hand, sub-classes of TCs (two types of horizontal system cells—HSN and HSE, and three types of vertical system cells—VS2, VS3 and VS4) did not separate into different clusters according to their *R* values (**Fig 3B**). This was not surprising since TCs were previously characterized in detail using morphological models and shown to have similar inner branch rules even though their spanning areas are easy to distinguish [17]. In all TC classes, TPs were more regularly distributed than BPs and all *R*_*TP*_ values were close to *1*, indicating configurations close to complete spatial randomness.

**Fig 4:**
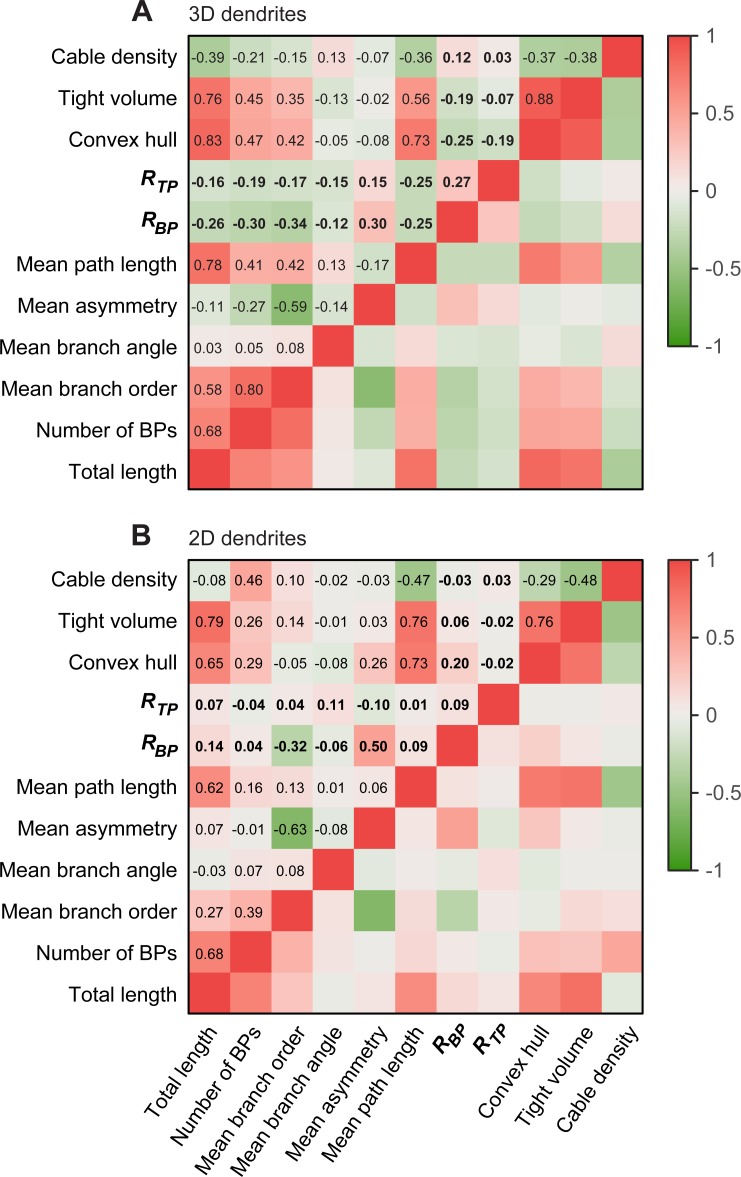
Correlation matrix between *R* values and other branching statistics. **A.** Correlation matrix of *R*_*BP*_ and *R*_*TP*_ with other typical branching statistics (bold) in the lumped 3D cells from **Fig 2A**. **B.** Similar correlation matrix but using the 2D cells of **Fig 2B**.

We also tested if the average distance to the NN of BPs and TPs of each individual cell corresponds to that of a uniform random pattern. The p-values for the tests were computed using the simulations of Poisson point cloud instances with the observed number of points, generated with our MC based approach. Considering 2D cells, da neurons showed a strong tendency to clustering in both their BPs and TPs: in 97.06% and 79.41% of the cases we rejected that the distribution of BPs and TPs, respectively, was uniform random in favor of a clustered distribution. All 3D cell types, except for dentate granule cells, showed rejection of a random distribution in their BPs with high confidence, in favor of a clustered distribution. In general, only few 2D or 3D cell types hinted to regular BP or TP distributions (see **S1 Table** for detailed results of all analyzed cell types). The statistic *R* for BPs and TPs is therefore a useful measure to distinguish between cell classes and characterize the relationship between dendritic tree structure and input architecture. However, it remains to be shown that the use of this local statistic in dendritic morphology is not simply an altered version of another traditional branching statistic. In order to test this and to check whether the input architecture as measured by *R* is reflected in other branching statistics, we computed the pairwise correlations between *R* and other commonly used statistics in 3D (**Fig 4A**) and 2D (**Fig 4B**) dendrites. We found that *R*_*BP*_ and *R*_*TP*_ do not have strong correlations with other typical branching statistics of dendritic trees in both cases. Since *R*_*BP*_ and *R*_*TP*_ were different in distinct cell types and were weakly correlated with other branching statistics, we postulate that these measures are a useful addition to the collection of branching statistics used to classify dendritic morphology.

### Morphological models with a given value of *R* for synaptic inputs

**Fig 5:**
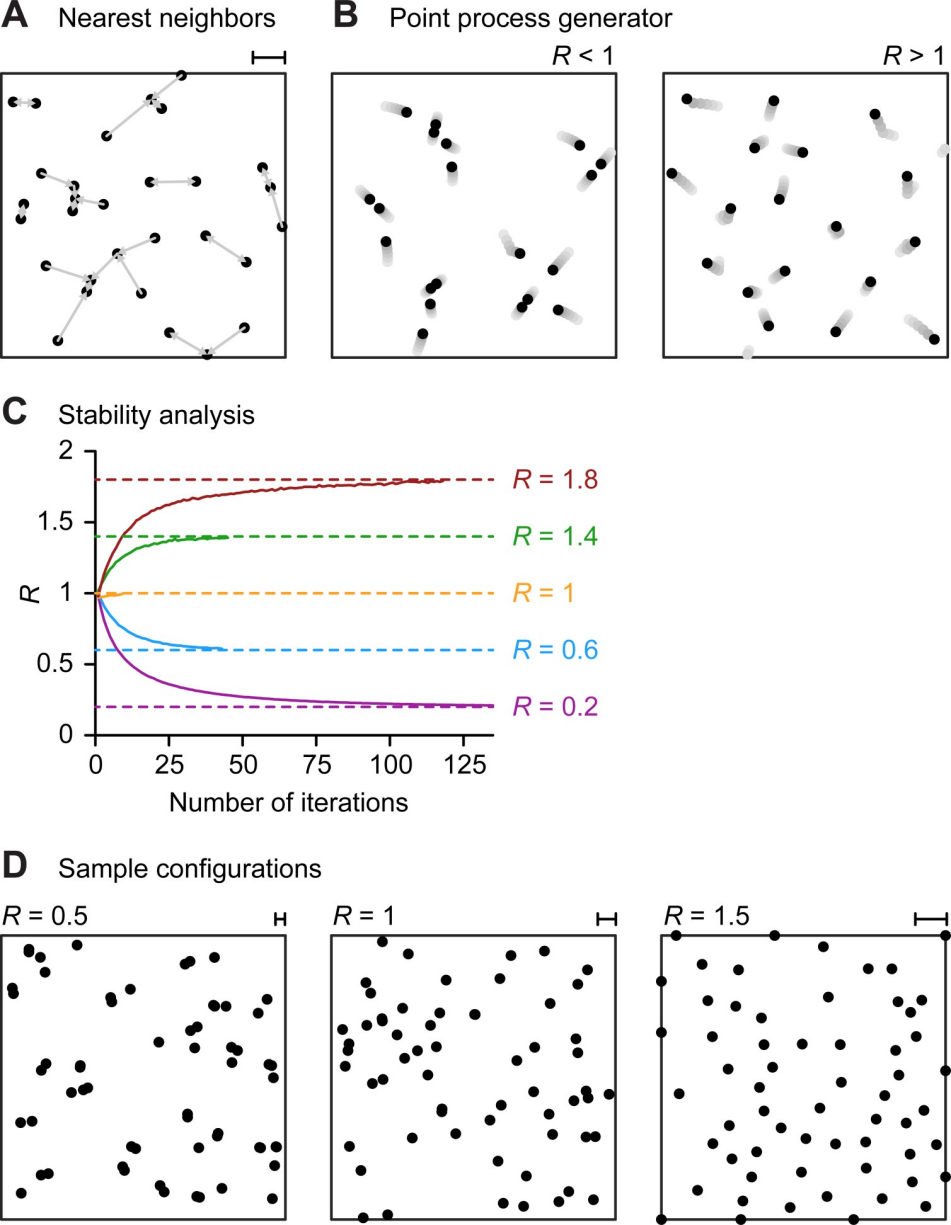
Point pattern generator for target NN distance. **A.** Illstration of average NN distance for uniform random Poisson distribution of *30* points and arrows indicating the individual NN. Scale bar (upper right) shows average nearest neighbor distance ${\bar{r}}_{0}$. **B**. Movements of *20* such points in the first *5* iterations (from light grey to dark grey) of our point pattern generator towards a clustered (left) and a more regular (right) pattern. **C**. Number of iterations required in our algorithm to obtain different values of *R* (*0*.*2* –purple, *0*.*6* –blue, *1* –yellow, *1*.*4* –green and *1*.*8* –brown) from an initial point cloud with *1*,*000* random points. Dashed lines show target *R* values. The algorithm stops when the target value of *R* is reached. **D.** Sample distributions of *50* points for *R* = 0.5 (left), *R* = 1 (middle) and *R* = 1.5 (right). Scale bars show average nearest neighbor distance ${\bar{r}}_{0}$.

In order to estimate how clustered or regular distributions of synapse locations co-depend with the clustering of BPs and TPs, we generated morphological models targeting different sets of input points with specified values *R*_*Input*_ of their statistic *R*. This process required a target point cloud generator for specified *R* values. To obtain a wide range of sets of target points with specific *R* values and number of target points, we started with a set of points obtained from a uniform random distribution; we then iteratively moved the input points until the point cloud reached a set target *R* value (see Methods and **Fig 5**). Dendrites were considered as tree structures connecting these target points [7,15].

**Fig 6:**
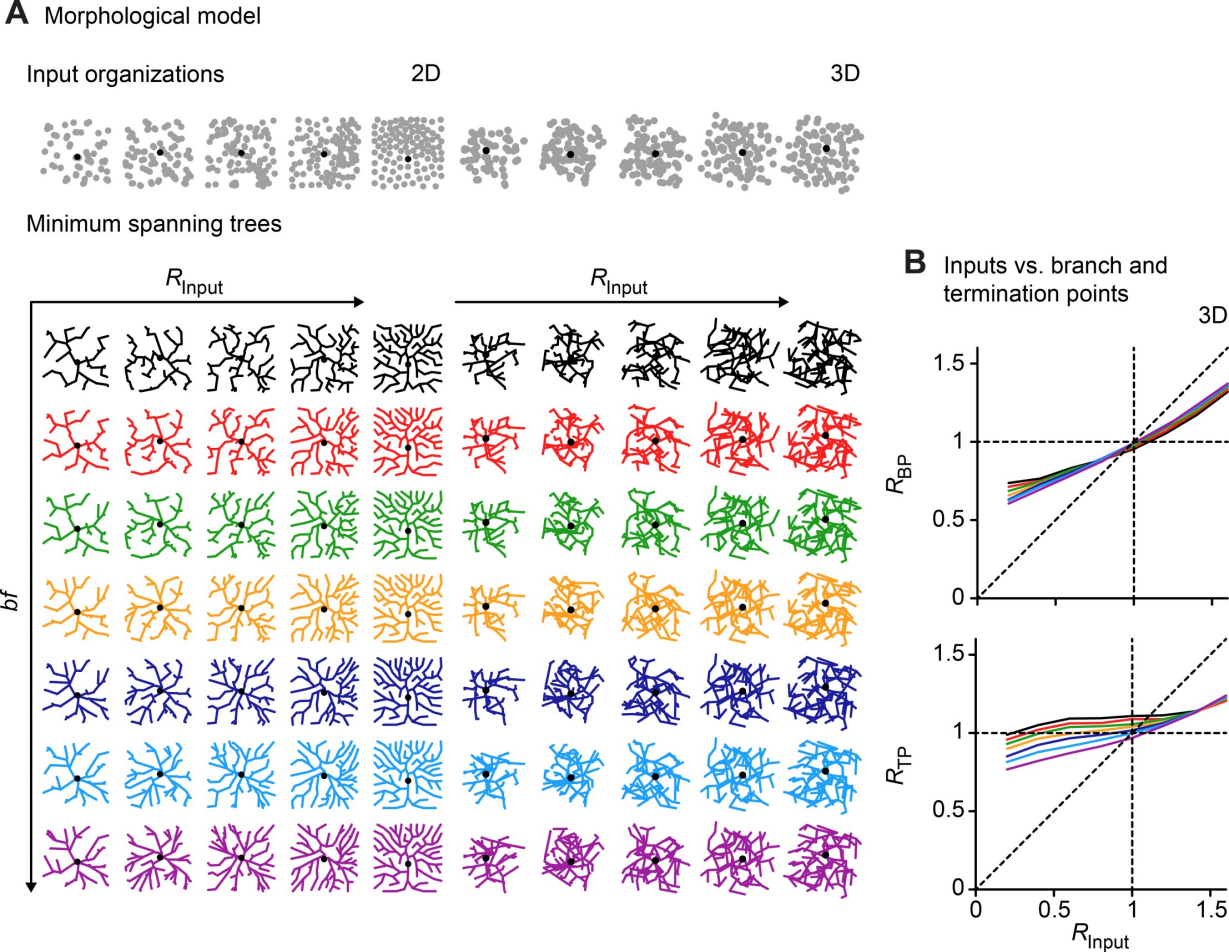
Relation between NN in the input distribution and in the dendrite’s BPs and TPs. **A.** Top row: *100* points (grey) distributed in a square (left, 2D) and cubic (right, 3D) area with different values of *R* (*0*.*2*, *0*.*6*, *1*, *1*.*4* and *1*.*8* from left to right each) using our point pattern generator. Synthetic trees to optimize wiring for different *bf* values are shown below each point cloud (from *0*.*2* to *0*.*8* in *0*.*1* increments from top to bottom), in 2D on the left for better visualization and in 3D on the right. Black dots indicate the root of the dendrite. **B.** Relationship between *R*_*Input*_ and *R*_*BP*_ as well as *R*_*TP*_ for all 3D morphological models with *N = 100* (average of *100* trees for each analyzed *R*_*Input*_ value). We obtained similar results in the 2D case as well as for higher point densities (*N > 100*).

We computed synthetic branching structures connecting the target points with minimal resources using the extended minimum spanning tree (MST) algorithm [7]. The MST connecting the target points minimizes at the same time both total cable length and the path length from any point along the tree to the root, using a parameter which weighs the two costs via a balancing factor (*bf*; see Methods). **Fig 6A** shows planar and 3D sample trees obtained from connecting *100* target points in a *200 μm x 200 μm* square and *200 μm x 200 μm x 200 μm* cube with different values of *R*_*Input*_, using the extended minimum spanning tree for different values of *bf*. Tree structures on sets of target points connected with the MST algorithm were previously studied for periglomerular neurons in the olfactory bulb and they were shown to approximate numbers and features of actual synapses [15]. Studying the relationship between target points and MSTs will therefore be informative about the relationship between synapses and dendritic trees.

**Fig 7:**
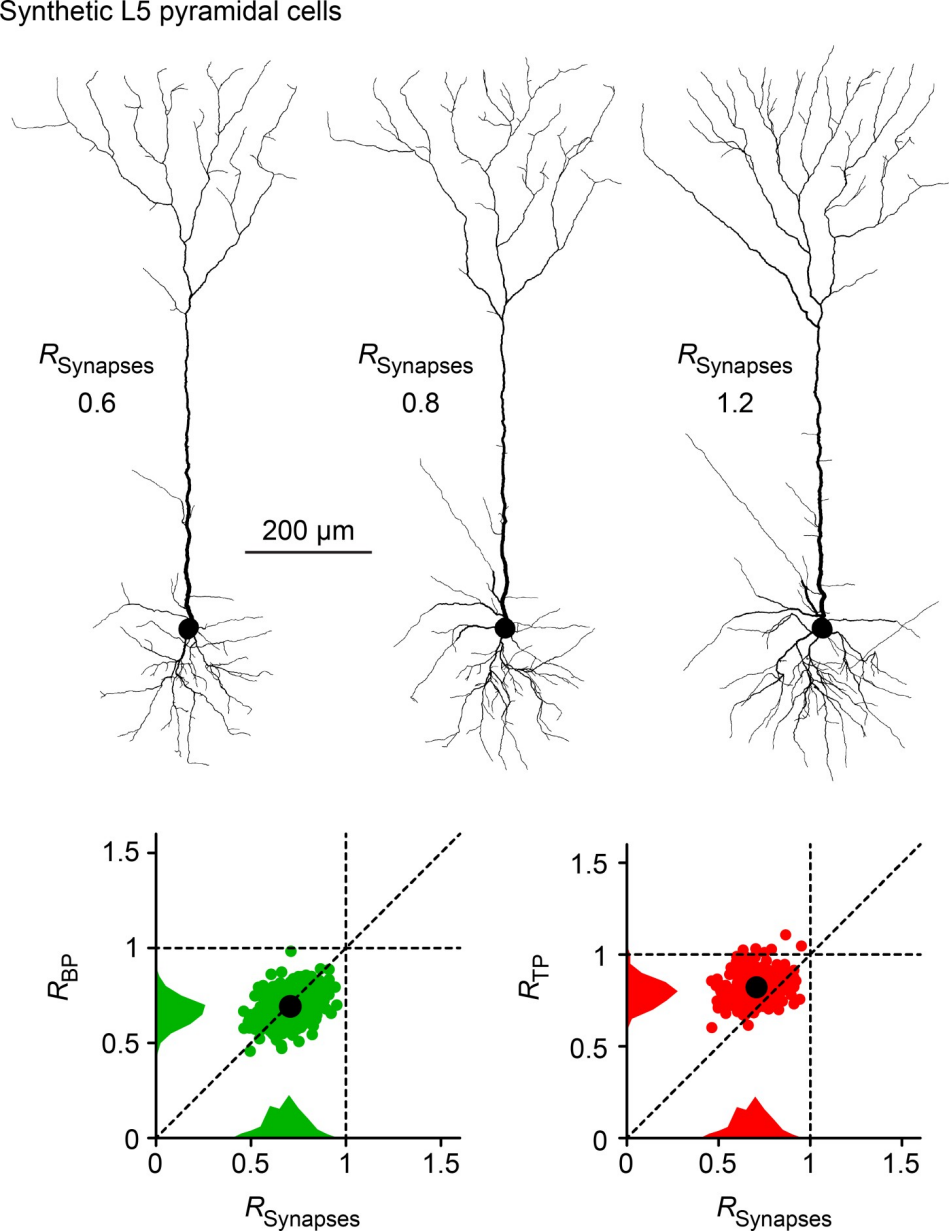
Relation between *R*_*Input*_, *R*_*BP*_ and *R*_*TP*_ in a detailed morphological model of L5 cortical pyramidal cells. 3 sample synthetic cortical Layer 5 pyramidal cells grown based on the assumption of optimal wiring. The respective target locations were chosen to have *R*_*Input*_ values of 0.6, 0.8 and 1.2. Lower panels show *R*_*BP*_ values in a dataset of synthetic Layer 5 pyramidal cells from a realistic normal distribution of *R*_*Input*_ *= 0*.*7 ± 0*.*1* in green and *R*_*TP*_ in red. Averages are shown as a larger black dot and histograms are collapsed onto each axis for the respective measures.

We generated morphological models on different sets of 2D and 3D target points with specific values of *R*_*Input*_ and found that with higher *R*_*Input*_ the trees became denser and the branches were more regularly distributed. This can be clearly observed in the 3D sample trees of **Fig 6** as *R*_*Input*_ increases. As might be expected, more regularly distributed inputs generally resulted in more regular branching structures (**Fig 6B** for 3D cases in **Fig 6A**). Compared with the input point distributions, BPs and TPs describing the dendritic geometry were more regularly distributed in all cases where *R*_*Input*_ *< 1* and were slightly less regular than the input point distribution in cases where *R*_*Input*_ *> 1*. Furthermore, the spatial organization of BPs and TPs was clearly different: in line with reconstructions of real dendritic trees, *R*_*TP*_ values were consistently closer to 1 while *R*_*BP*_ followed *R*_*Input*_ more faithfully. We obtained similar relations between *R*_*Input*_, *R*_*BP*_, and *R*_*TP*_ for different numbers of points. *R*_*BP*_ is therefore most likely a better estimate for the regularity of a neuron’s underlying synaptic input organization. To show that this was also the case in more detailed morphologies we used a morphological model from layer 5 cortical pyramidal cells [7] and obtained similar results in a realistic range of *R*_*Input*_ *= 0*.*7 ± 0*.*1* (**Fig 7**).

**Fig 8:**
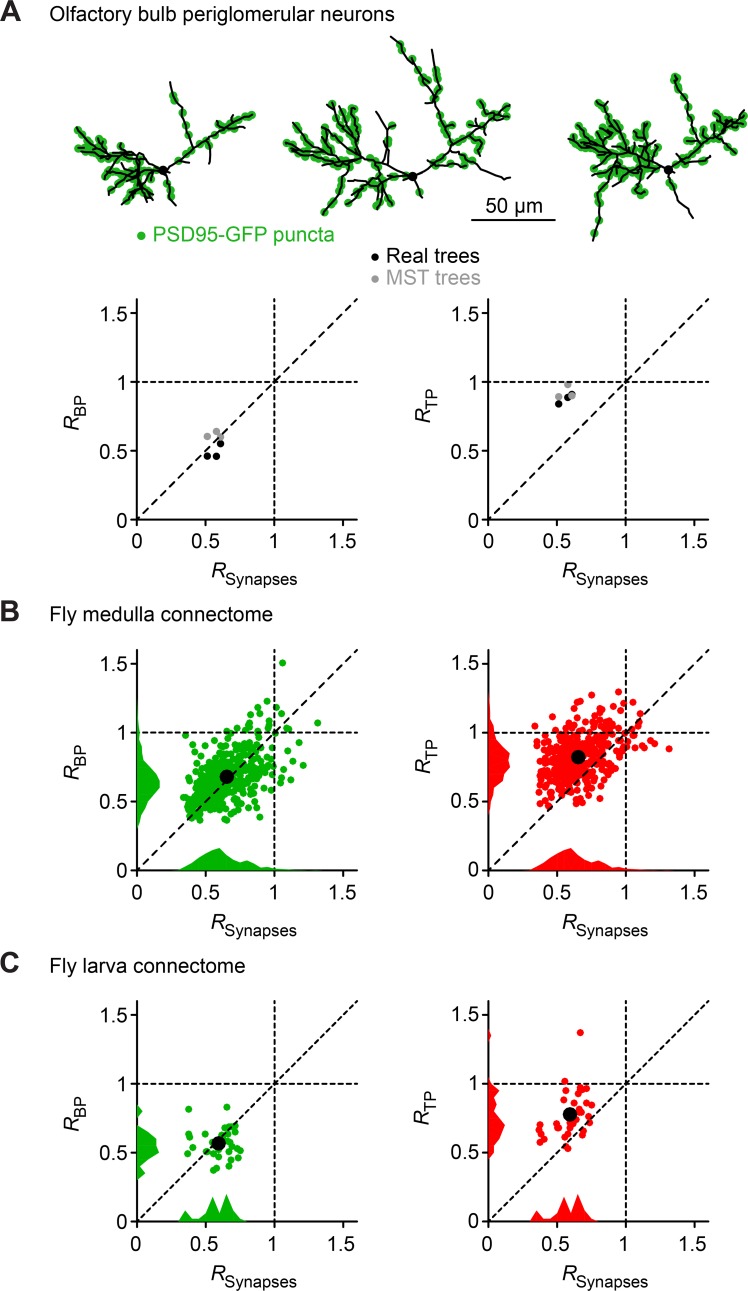
Relation between *R*_*Input*_, *R*_*BP*_ and *R*_*TP*_ in real cells. **A.** *R*_*Input*_ from actual synapse locations measured by PSD-95-GFP puncta in adult born periglomerular neurons of the olfactory bulb [30] in comparison with *R*_*BP*_ (left) and *R*_*TP*_ (right) in the corresponding trees. Dendritic morphologies with synaptic puncta (green) are shown (modified from [15]). The results are shown for the actual dendritic reconstructions (black dots) and for MSTs grown using the synapse locations as target points (grey dots). **B**. *R*_*BP*_ vs. *R*_*Input*_ in the complete dataset from a previously published connectome of neighboring fly medulla columns [31] in green and *R*_*TP*_ in red. Averages are shown as a larger black dot and histograms are collapsed onto each axis for the respective measures. **C**. Similar presentation as in B but for the fly larva dataset from [32].

We compared this result with one biological dataset where synapse locations and dendritic reconstructions were available (**Fig 8A**), specifically for the aforementioned adult-born periglomerular neurons of the olfactory bulb [15,29,30]. Interestingly, the results for both actual reconstructions and MSTs constructed on the actual synapse locations were in line with our simulations from this study. These results indicate that *R*_*Input*_ could be estimated using *R*_*BP*_ whereas termination points in dendrites and MSTs are more randomly distributed, characterized by a value of *R*_*TP*_ close to 1. We then compared *R*_*Input*_, *R*_*BP*_, and *R*_*TP*_ in two connectome datasets from the fly (**Fig 8B and 8C**) [31,32]. Using serial electron microscopy it has increasingly become possible to obtain data of morphological reconstructions and the exact position of synapses between the same cells. Interestingly, we found the predictions from our model to be validated by these two datasets with *R*_*BP*_ and *R*_*Input*_ values being more similar and *R*_*TP*_ being closer to 1. Overall, we believe that constructing synthetic versions of real dendritic trees with more detailed morphological models would be useful for inferring the underlying spatial organization of the synaptic inputs. This will provide invaluable predictions for connectome and large-scale neural circuit analyses.

**Fig 9:**
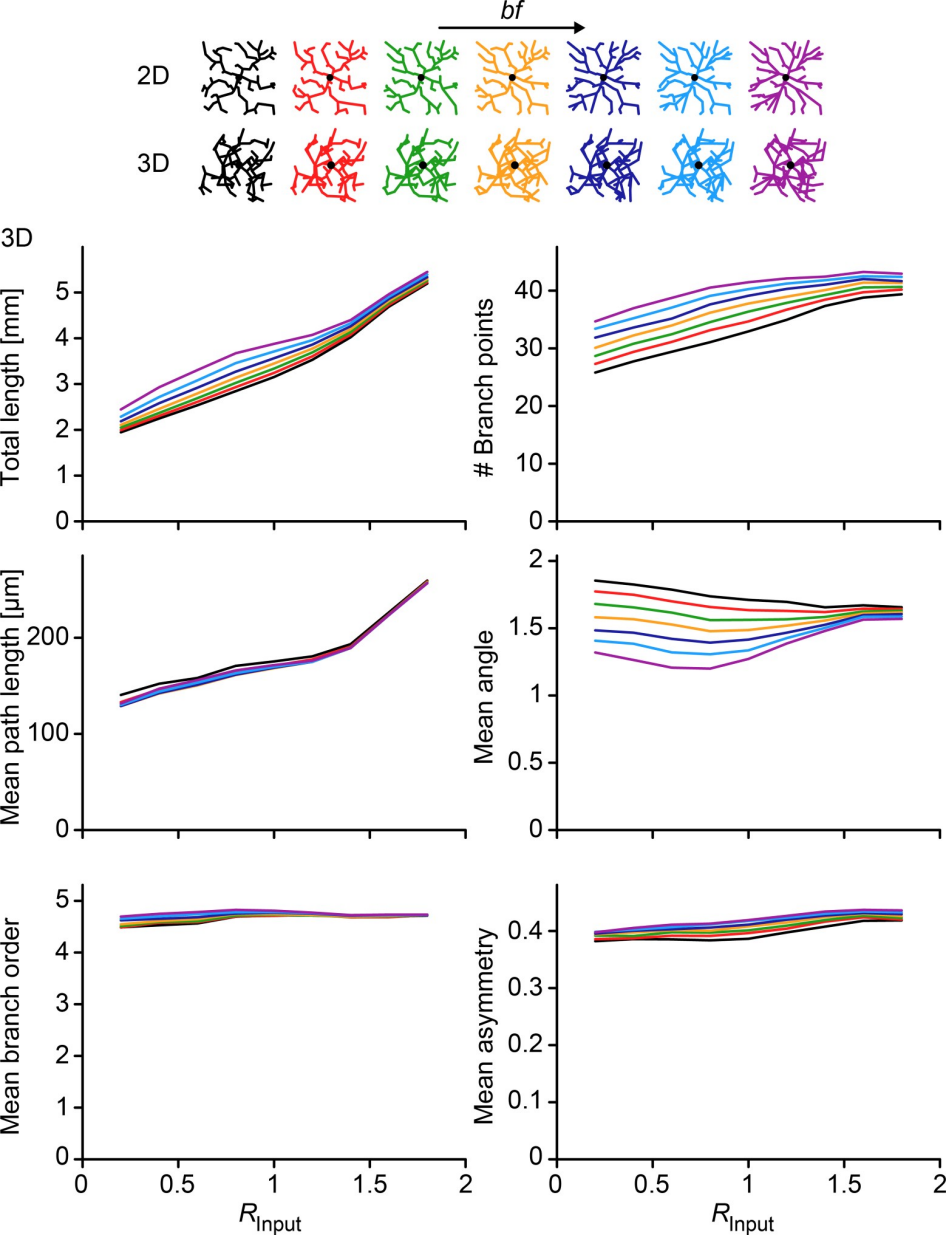
Relation between *R*_*Input*_ and dendritic branching statistics in the morphological model. Total dendrite length, number of branching points, mean path length from any point to the root, mean angle (in radians) at branch points, mean branch order for any point on the dendrite and mean asymmetry at the branch points for all cases in **Fig 6**. Colors indicate the same values of *bf* in the morphological model with sample morphologies plotted from **Fig 6** at the top of the figure.

**Fig 10:**
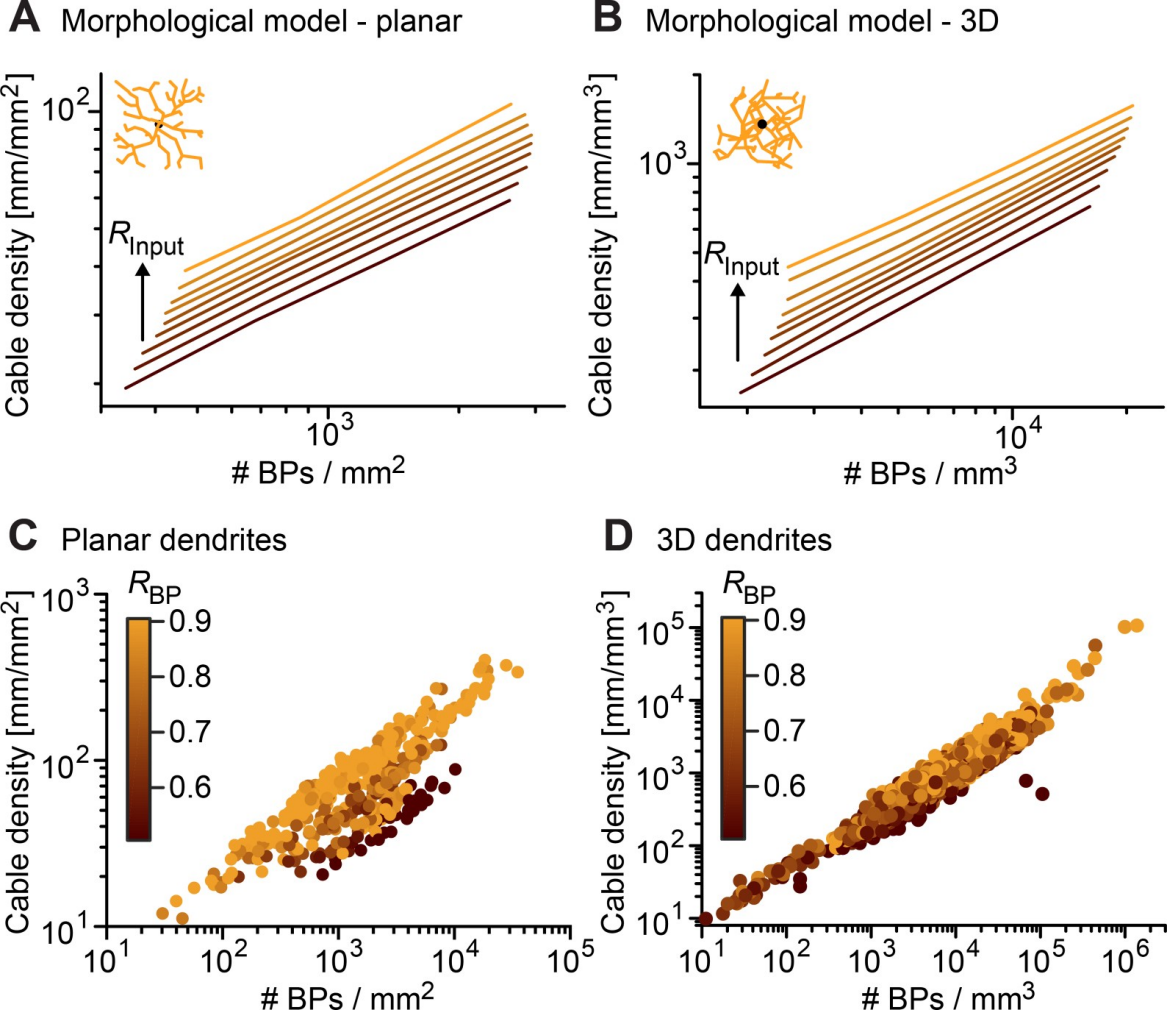
Scaling relation of cable density and BP density for real and model cells. **A, B**. Cable density vs. BP density in the 2D and 3D morphological model of Fig 6 (*bf* = 0.5). Increasing *R*_*Input*_ (0.2 to 1.8 in steps of 0.2) is indicated with lighter color, same values as in **Fig 6**. Results were qualitatively similar for different *bf* values. **C, D**. Similar plots for the 2D and 3D reconstructions from **Fig 2**. Here, *R*_*BP*_ values are indicated with color (see color bars).

Apart from the relations between *R*_*Input*_, *R*_*BP*_, and *R*_*TP*_, it is interesting to study the relation of *R*_*Input*_ with branching statistics typically used to characterize dendritic trees (**Fig 9**). As was the case for *R*_*BP*_ and *R*_*TP*_ in real dendritic tree reconstructions, *R*_*Input*_ was weakly correlated with other branching statistics, suggesting that input architecture is not well captured by traditional branching statistics whereas *R*_*BP*_ and *R*_*TP*_ would be useful measures for this and to classify dendritic morphology accordingly. However, both total length and number of branch points increased reliably with *R*_*Input*_, requiring the minimum spanning tree to use more cable to connect the points that are more widely spread and more branches to reach out to all distributed inputs in space. This correlation clearly affected the scaling behavior that was previously observed between number of inputs and total length as well as between number of inputs and number of branch points [15]. Here, the previously reported 2/3 power between these measures was not affected by *R*_*Input*_, but a clear increase in total length was observed as an offset in the relationship (**Fig 10**). MST-based dendrites connecting target points with an increased *R*_*Input*_ required much more cable length.

## Discussion

We have presented here a new branching statistic for dendrites, the regularity index *R*, which is based on the average nearest neighbor (NN) distance between branch, termination or input points of a given dendritic tree, capturing the regularity of their respective distributions. Specifically, *R* is defined as the ratio of the observed average NN distance to the one expected in a matching random point cloud. This makes *R* independent of the absolute scale of the dendritic arbor, but rather captures the clustering characteristic of the branch and termination points and allows for comparison of cells of different sizes. We found that the measure allowed to distinguish dendritic trees from different cell classes for which the local statistics of the spatial input distribution differed. The values of *R* computed for the sets of branch points (*R*_*BP*_) and termination points (*R*_*TP*_) of reconstructions of real dendritic trees correlated little with most other commonly considered branching statistics, indicating that these measures provide new descriptive power for dendritic trees that was not captured by existing measures.

Using morphological models, we then found that, in the range of observed *R*_*BP*_ and *R*_*TP*_ values in real cells, overall *R*_*BP*_ values were good predictors of the input distribution (*R*_*Input*_) while *R*_*TP*_ values were generally closer to 1, implying more randomly distributed termination points in dendritic trees. We also showed that more regular input distributions with higher values of *R*_*Input*_ showed increased dendritic total length in the model. An analogous increase in total length was observed in real dendrites with increasing *R*_*BP*_. Input targets can loosely be compared to synaptic inputs for certain cell types such as the periglomerular neurons of the olfactory bulb in Fig 8A [15]. While this indicates a specific link between the input organization and dendritic morphology it by no means implies a causal relation between input locations and resulting dendrite growth. Rather, dendrites and axons organize to implement possible connectivity patterns between neurons and specific dendritic morphology could constrain the space of potential synapses. However, the precise biological processes are not yet known.

On the one hand, the correlation between the synapse distribution and *R*_*BP*_ can be a direct consequence of optimal wiring since minimum spanning trees shows the same correlation as real dendrites. But it is not known how the biological growth process leads to such optimal wiring. On the other hand, the precise distribution of branches within the range of optimally wired dendrites can vary substantially and the details of the growth process can lead to these differences in morphology. Increased self-avoidance of branches during dendrite development through molecular mechanisms such as Dscam would for example most likely lead to larger values of *R* [33]. Increased localized branching due to some accumulation of molecular cues could in the other extreme lead to clustered branches in mature dendrites leading to *R* values lower than 1. Overall, we expect the proposed measure *R* to be able to better predict these type of local features of the input organization of given dendritic tree types compared to other existing branching statistics. As more realistic morphological models based on minimum spanning trees become available, as is the case for example for TCs [17] and dentate gyrus granule cells [18], this information can be further refined.

The expected NN distance of a uniform random distribution used for calculating *R* has traditionally been obtained analytically, assuming infinitely many points in an unbounded volume. Yet, in practice all of our volumes *V* containing a given point cloud *C* were finite and bounded resulting in strong biases and edge effects if *R* is estimated in a naïve way (see Methods). Our approach to remedy these adverse effects was to use Monte Carlo (MC) simulations to predict the expected NN distances of uniform distributions numerically, using instances of Poisson point clouds. This procedure furthermore allowed for the specification of confidence intervals for the estimated values of *R* (**S2–S4 Figs**). We found that confidence intervals were mostly dependent on the number of points in the point cloud (decreasing as the number of points increases) and only to a lesser extent on the shape of its supporting volume. For small numbers of points (*N* < 20), the confidence intervals were large, making the estimated values of *R* less reliable (**S3 Fig**). Our MC based approach will be useful in further studies beyond the scope of dendritic morphology since point processes in any type of finite volumes will necessarily exhibit similar important boundary effects.

There are several ways in which the measure *R* could be generalized. First of all, for simplicity we assumed point clouds with uniform homogeneous densities when computing *R*. This can be extended to non-homogenous cases by studying local estimates of point densities. This would lead to a localized version of measure *R*. Secondly, we only considered one nearest neighbor per point. This allows for point clouds with different global clustering behavior to get assigned the same value of *R* (e.g. a point cloud where all points have the same coordinates and a point cloud where pairs of points have the same coordinates both get assigned a value of *R = 0*). But the statistic *R* can easily be extended to consider neighborhoods of higher order, containing the *k* nearest neighbors for each point, *k* ≥ 2. Furthermore, other quantities commonly used for the analysis of spatial point patterns such as the cumulative distribution function of the nearest-neighbor distances, known as G function, Ripley’s K function [34] and related quantities can be used. These extensions are subject of future studies. If neighbor distances are used to define simplicial complexes on a given point cloud, and the resulting complexes are examined using methods from algebraic topology, this leads to techniques used in topological data analysis [35]. Related techniques most recently also were proposed as methods to examine dendritic structures [36].

Overall, we presented a new statistic, the regularity index *R*, for dendrites that allows to relate the morphology of a neuron with the specific connectivity that it implements. It has low correlation with most commonly used statistics of dendritic branching and is extendable in several ways, providing a useful new statistic for the classification of dendritic trees.

## Methods

The analysis was performed with the TREES toolbox [7] (*www*.*treestoolbox*.*org*), an open-source software package for MATLAB (Mathworks, Natick, MA). Functions *r_mc_tree* to calculate *R* values in existing trees, as well as *PP_generator_tree* to generate point distributions with given *R* values were newly implemented and are now available in the TREES toolbox package.

### Average nearest neighbor ratio *R*

The average nearest neighbor (NN) ratio $R={\bar{r}}_{0}/{\bar{r}}_{E}$ compares the observed average NN distance ${\bar{r}}_{0}$ between a set of *N* points with the expected average distance ${\bar{r}}_{E}$ between nearest neighbors under the assumption of a uniform random distribution (with the same number of points covering the same total area or volume). This approach was first described in [20,37].

*R* provides a measure of the clustering of the points in a point cloud *C*. Concretely, the closer the points are to a random (Poisson) distribution, the closer to *1* the value of *R* becomes (as the values of ${\bar{r}}_{0}$ and ${\bar{r}}_{E}$ are more similar). Values of *R* less than *1* correspond to clustering (${\bar{r}}_{0}<{\bar{r}}_{E}$). When all points overlap (*R* = 0) the most clustered condition is reached. For values of *R* greater than *1*, nearest neighbors are further apart than it would be expected for a random distribution (${\bar{r}}_{0}>{\bar{r}}_{E}$). In 2D arrangements, the most dispersed situation is the one in which the points are spaced on a triangular lattice, yielding a value of *R* = 2.1491 [20]. The measure *R* has the advantage of being easily interpretable. For example, *R* = 0.5 indicates that nearest neighbors are, on average, half as distant as expected under random conditions (c.f. **Fig 5D**). Note though that *R* only considers pairwise NN distances and e.g. cannot distinguish the case in which all pairwise NN distances are 0 (pairs of points have the same coordinates) from a fully clustered situation (all points of the point cloud have the same coordinates).

Formally, for a finite point cloud *C*, i.e., a set of *N* points, the average NN distance is

$${\bar{r}}_{0}=\frac{1}{N}\sum_{\begin{array}{c} i=1\\ i\neq j \end{array}}^{N}min\{d_{i,j}\},$$

where *d*_*i*,*j*_ denotes the Euclidean distance between the *i-*th and the *j-*th point in *C*. This is the numerator in the definition of $R={\bar{r}}_{0}/{\bar{r}}_{E}$. The denominator in *R* is the expected NN distance ${\bar{r}}_{E}$ for a Poisson process that can be analytically computed as ${\bar{r}}_{E}=1/2\sqrt{\rho }$ in the 2D case and as ${\bar{r}}_{E}=\Gamma (4/3)/\sqrt[{3}]{4\pi \rho /3}$ in the 3D case, where Γ(∙) is the gamma function and *ρ* is the point density, i.e., the mean number of points per unit area or volume *V*. For a uniform random distribution, an unbiased estimator of *ρ* is $\hat{\rho }=N/V$. Thus, to obtain the point density, an accurate estimate of the supporting volume *V* of the point cloud *C* is required.

### Computing the supporting volume of a point cloud using α-shapes

In order to estimate *R*, a volume *V* supporting a given point cloud *C* needs to be estimated. The most common way to do this is to use the convex hull of *C*. Yet, with this choice the supporting volume is overestimated if it is non-convex, which results in incorrect values of *R* (see for examples **S1 Fig**). Better estimates of *R* were obtained using α-shapes. α-shapes were devised to characterize the shapes of point clouds and can be seen as an extension to the notion of a convex hull [38,39]. Formally, to any given finite point cloud *C* in 2D or 3D Euclidean space a one parameter family of curves or surfaces *S*_*α*_ called α-shapes can be constructed, with *α ϵ* [0,∞]. By construction, *S*_*∞*_ corresponds to the convex hull and *S*_*0*_ to the point cloud itself. For any finite *C*, *S*_*α*_ is a finite set and a smallest value *α*_*0*_ exists (called critical value of *α*) such that $S_{\alpha_{0}}$ is connected and contains all points of *C*. Furthermore a smallest value *α*_*k*_ < ∞ exists for which $S_{\alpha_{k}}=S_{\infty }$. The α-spectrum of *C* is defined as the monotonically increasing, finite sequence of values (*α*_*i*_)_0≤*i*≤*k*_, 0 ≤ *α*_*i*_ < ∞, *α*_*i*_ < *α*_*i*+1_ for which each $S_{\alpha_{i}}$ is a distinct α-shape and the shapes do not change between two consecutive values *α*_*i*_,*α*_*i*+1_. To compute what we call a “tight hull” around a point cloud *C* we selected the center point *α*_*k*/2_ of the α-spectrum, for which we rounded the index *k*/2 to the next integer value. Especially for point clouds with non-convex supporting volumes, this yielded much better estimates of the true volume and thus less biased estimates of *R*. See **S1B and S1C Fig** for an example of the convex hull compared to the tight hull of point clouds with non-convex support, and the resulting values of *R*.

### Boundary effects and Monte Carlo approximation of *R*

After estimating a supporting volume *V* using α-shapes as described in the previous section, naïve calculations of *R* still yielded biased results due to boundary effects. This is due to points in *C* for which the distance to the boundary of *V* was smaller than the average NN distance ${\bar{r}}_{0}$ in *C*. As the calculation of ${\bar{r}}_{E}$ assumes that balls of radius ${\bar{r}}_{E}$ surrounding all points are always completely contained in *V*, *R* was overestimated. Two proposed techniques correcting for such boundary induced biases are the so-called toroidal edge correction and the border area edge correction. The first one removes boundaries by transforming a (bounded) rectangular study region into a torus by identifying opposing edges. The second one specifies a buffer zone around the boundary of the study region and uses the part remaining in the middle as the new study region. Points in the buffer zone are used only to take measurements of points (NN distances in our case) that are within the new study region and are further discarded. Yet, both techniques have their drawbacks: the toroidal edge correction cannot be used for non-rectangular regions, as is the case of dendrites, and the border area edge correction discards a large number of available points, which makes it inappropriate for many dendrites for which the number of points was not large enough. Moreover, analytical bias corrections were also derived [28], but those require convex planar surface areas as supporting volumes.

Since most dendritic arbors form non-convex areas and volumes (**S1A Fig**) and many of them do not have a high number of BPs and TPs, instead of computing ${\bar{r}}_{E}$ analytically from an estimate of the point density and using an edge correction technique, we used a Monte Carlo (MC) simulation approach to estimate ${\bar{r}}_{E}$. For a point cloud *C* consisting of *N* points contained in a volume *V*, we first computed ${\bar{r}}_{0}$ as the observed mean NN distance in *C*. We then sampled *M* = 100 uniform random point clouds within *V*, each containing *N* points. Importantly, we scaled each sampled point cloud so that its supporting volume (again computed using α-shapes) matched *V*. When this process, which we call volume correction, was not performed the estimates of *R* values were positively biased, especially for small point clouds (**S2 Fig**). For each of those point clouds we then computed the average NN distance, ${\bar{r}}_{E_{i}},\;i=1,\ldots ,M,$ and obtained an estimate of ${\bar{r}}_{E}$ as the mean of the ${\bar{r}}_{E_{i}},\;i=1,\ldots ,M,$ values of the simulations. No edge corrections were necessary since all the average NN distances were biased by the same edge effects. To check the correctness and convergence properties of this approach, we generated point clouds with known *R* values and compared them to the *R* values estimated from our MC based method (**S4 Fig**). In each MC iteration we additionally estimated confidence intervals $\left[c_{i}^{-},c_{i}^{+}\right]$ for ${\bar{r}}_{E_{i}},i=1,\ldots ,M$ with confidence level 1 − *α* using 1,000 bootstrap samples. We then obtained the corresponding confidence intervals [*c*^−^,*c*^+^] for ${\bar{r}}_{E}$ by computing the sample mean of the set of $c_{i}^{-}$ and $c_{i}^{+},i=1,\ldots ,M,$ to obtain *c*^−^ and *c*^+^, respectively. The confidence interval [*c*^−^,*c*^+^] for ${\bar{r}}_{E}$ in turn leads to the statistic *R* as $\left[\frac{{\bar{r}}_{0}}{c^{+}},\frac{{\bar{r}}_{0}}{c^{-}}\right]$. Throughout this study we used *α* = 0.05. We observed that the confidence intervals were mainly influenced by the number *N* of points in the point cloud and to a much lesser extent by the shape of the supporting volume *V*. We assessed this by computing confidence intervals both for planar sample configurations with known *R* value (**S2 Fig**) in a square area and for the 3D cell classes considered in this work (**S3 Fig**).

### *R* values for dendrites from NeuroMorpho.Org

To evaluate the measure *R* on real cells, we obtained a number of reconstructions of dendritic trees from NeuroMorpho.org [40], Version 7.0 (released on 09/01/2016) using the TREES toolbox [7]. Specifically, we chose reconstructions belonging to eight well-known cell classes for our investigations, namely cortical pyramidal cells, hippocampal pyramidal cells, dentate granule cells, motoneurons, retinal ganglion cells, cerebellar Purkinje cells, fly larva dendritic arborization (da) neurons and fly Lobula Plate tangential cells (TCs). The first four classes were 3D cells and the last four classes were 2D. For selecting the reconstructions, we obtained all reconstructions from NeuroMorpho.org that were classified as either having "moderate" or "complete" reconstructions of their dendritic trees and belonged to the control group (to exclude mutant cells). We then grouped all reconstructions by archive and sorted out archives that contained poor reconstructions by manual visual inspection as well as archives containing one cell only. This left us with a number of reconstructions of each cell type, denoted in parentheses in the following list: cortical pyramidal cells (*3784*), hippocampal pyramidal cells (*399*), dentate granule cells (*154*), motoneurons (*82*), retinal ganglion cells (*322*), cerebellar Purkinje cells (*15*), fly da neurons (*68*), fly TCs (*55*). After downloading the reconstructions in the SWC format, these were read into and pre-processed using the TREES toolbox. For each reconstruction, this process involved deleting the soma and the axon if present (function *delete_tree* for the SWC regions *1*, *2*, *5* and *10*) and then re-joining the parts of the tree if the deletion operation yielded several roots (function *catx_tree*), followed by a final removal of higher order multifurcations (function *repair_tree*). This process was not necessary for fly TCs that were available with the TREES toolbox [17]. Da neurons were furthermore subdivided into da class I-IV cells and TCs into horizontal system northern (HSN) cells, horizontal system equatorial (HSE) cells, and vertical system (VS2, VS3, and VS4) cells (**Fig 3**). For each tree we then computed a number of statistics: total dendrite length, number of BPs, mean branch order of BPs and TPs, mean branch angle, mean asymmetry [41], mean path length, *R* for the set of BPs (*R*_*BP*_), *R* for the set of TPs (*R*_*TP*_), volume of the convex hull, volume of the tight hull and cable density as total length per volume in the tight hull. The tight hulls as well as their volumes needed for estimating *R* were computed using α-shapes as described previously (function *boundary*).

### Point pattern generator with target *R*

In order to study a wide range of different spatial input organizations in the morphological model we implemented a procedure for obtaining point clouds with specified *R* values. First, we generated a number *N* of random points within a square or cube. We then iteratively estimated the *R* value using our MC method and moved each point in the direction of or away from its NN, depending on whether the target *R* was smaller or greater than the current *R*, respectively, (**Fig 5B**) until the target *R* value was reached. The shift was proportional to the difference between the current *R* value and the target *R*, i.e., the closer the values of both, the smaller the movements. **Fig 5C** shows the number of iterations required for our algorithm to reach different values of *R*, from highly clustered (*R* = 0.2) to highly regular (*R* = 1.8) given *1*,*000* initial points. We obtained very similar results for different numbers of points.

### Morphological models connecting points with different *R* values

Using the point pattern generator described above we generated a large number of point clouds in 2D or 3D spaces. Planar arrangements were fixed to *200 μm x 200 μm* and 3D arrangements were set to *200 μm x 200 μm x 200 μm*. A large variety of number of points (*50–400* points) and *R* values (*0*.*2–1*.*8*) were computed. We subsequently computed morphological models based on optimal wiring principles that connected these point clouds. Optimal wiring of the underlying point cloud was implemented using a minimum spanning tree as described in [7] using the algorithms available in the TREES toolbox [7,42]. Briefly, optimal wiring minimizes both total cable length and the path length from any point along the tree to the root, using a balancing factor *bf* to weigh the second cost (that is: *total cost* = *cable length cost* + *bf* ∙ *path length cost*). For *bf* = 0 the algorithm only seeks to minimize the total cable length while for large *bf* it seeks also to minimize the length of the connections from the root to any point. Values of *bf* greater than 0 and less than 1 represent a mixture of the two objectives that are realistic for real dendrites. As a further constraint, we did not allow multifurcations (more than two daughter branches at each BP) in the computed synthetic trees.

The minimization was achieved via a greedy minimum spanning tree algorithm [43]. We computed synthetic dendritic trees from all the point clouds, connecting the points to a root in the center and using *bf* values from *0*.*2* to *0*.*8*. We obtained *100* trees for each individual condition (point density, *R* and *bf* value). For each synthetic dendritic tree, *R*_*Input*_ of its target points was known (since the inputs were obtained using the point pattern generator), and we estimated *R* values of its BPs (*R*_*BP*_) and TPs (*R*_*TP*_) in order to study the relation between these measures. In addition, we analyzed the relation between *R*_*Input*_ and other branching statistics commonly used to describe dendritic morphology. Specifically, we studied the total length, the number of branching points, the mean path length from the root of BPs and TPs, the mean branching angle, the mean branching order and the mean asymmetry at the branching points of each synthetic dendritic tree. The asymmetry for each branching point was defined as the ratio of *v*1/(*v*1 + *v*2) for *v*1 < *v*2, where *v1* and *v2* are the counts of TPs in each of the two daughter branches. All statistics were computed using the TREES toolbox.

We also considered a possible exclusion zone around the target points due to the physical dimension of synapses that these might represent. This is an optional parameter *ε* in the *PP_generator_tree* function, a minimal distance between all points in the resulting point pattern. All morphological models from **Figs 6, 9 and 10** were recalculated with *ε = 0*.*5 μm* with very similar results (**S5 Fig**). The simpler results without considering volume exclusion are shown in the main manuscript. It is worth noting that real neurons grow in a packed tissue and volume exclusion also most likely plays a role in the growth process of the dendritic trees. However, simulating dendrite growth in the packed tissue was outside of the scope of this study.

### Morphological model of L5 cortical pyramidal cells

We used a model previously described in Cuntz et al., 2010 [7], to check whether the conclusions for simple abstract morphological models hold for more realistic morphologies. The construction pipeline has been well described previously but was adapted to rearrange the targets for apical, oblique and basal dendrites separately to match a given target *R*_*Input*_ using our new *PP_generator_tree* function. Only then were the targets connected to MSTs as described earlier. Synthetic morphologies (*N = 332*) were generated for **Fig 7** with *R*_*Input*_ values from a normal distribution around *0*.*7 ± 0*.*1*. The numbers were chosen to approximately simulate a distribution of dendritic parameters for cortical pyramidal cells (see Fig 2). Sample morphologies were generated with the same initial target point distribution altered to specifically match *R*_*Input*_ of *0*.*6*, *0*.*8* and *1*.*2* using *PP_generator_tree*.

### Connectome analysis

Data in **Fig 8** were obtained from three different datasets and processed in similar ways to obtain adequate values for *R*_*Input*_, *R*_*BP*_, and *R*_*TP*_. The same new TREES toolbox function *r_mc_tree* was used to calculate the *R* values separately on the synapse locations, branch points and termination points separately. The olfactory bulb dataset [29,30] was used similarly as previously [15]. The fly medulla dataset was obtained during a Hackathon at Janelia Farm Campus (https://github.com/janelia-flyem/SevenMedullaColumnConnectome) [31]. The fly larva dataset comes from an analysis of neurons of the peripheral nervous system at two larval developmental stages [32]. For the fly connectome datasets very tight hulls (α = 0.9) were used to avoid including lengthy axonal segments without synapses or branch points. Both inputs and outputs were included in the analysis since fly neurites contain both presynaptic and postsynaptic puncta on the same branches.

## Supporting information

S1 Fig Edge and boundary effects on the estimate of *R*. **A.** Example of a non-convex TC dendrite. Magenta shows the hull around BPs and cyan shows the hull around TPs. (Left) Convex hull. (Right) Tight hull. **B, C.** Examples of the implemented approximation to compute the *R* measure through a tight hull in 2D for an L-shape with *1*,*000* points (B), and in 3D for a double L-shape with *3*,*000* points (C). Expected *R* and volume *V* using the correct volume (computed analytically, red), using the convex hull (green) and using the tight hull (blue). *R*_*c*_ is the calculated value using our Monte Carlo (MC) approach within the tight hull to remove the remaining bias.
(TIF)

S2 Fig Confidence analysis when estimating *R* from *N* points. Single traces (left) and averages of 10 traces (right) for estimates of *R* without (top, blue straight lines) and with volume correction (bottom, red straight lines). Dots in all cases indicate the individual simulation results and shaded areas show the confidence intervals in each case. The grey shaded areas show the best possible confidence intervals calculated analytically. Each estimate of *R* was done with our Monte Carlo based estimator using 100 iterations.
(TIF)

S3 Fig Confidence analysis in dendrites of real 3D neurons. **A**. Confidence interval length for *R*_*BP*_ with respect to number of BPs for 3D cells. Confidence intervals decreased with number of BPs. **B**. Similar graph for the confidence in the *R*_*TP*_ measure. **C**. Estimated *R* values for BPs and TPs of 3D cells with confidence intervals. Horizontal axis shows estimated *R* value for sets of BPs, vertical axis estimated *R* value for sets of TPs of each cell. Each dot represents one cell, color coded by cell type. Horizontal and vertical whiskers indicate 95% confidence intervals for *R*_*BP*_ and *R*_*TP*_, respectively. Diamonds show average values and confidence intervals for each population.
(TIF)

S4 Fig *R* value estimation as a function of number of MC iterations. Estimated *R* values via MC for point clouds with known *R* in a square area with *N* = 50 points. Dashed lines show the true *R* values. The mean and standard deviation of *10* estimated *R* values are shown in green (*R* = 0.5), red (*R* = 1) and cyan (*R* = 1.5). Here we used from *50* to *100* Monte Carlo iterations to obtain each estimated *R*.
(TIF)

S5 Fig Morphological models considering volume exclusion. **A.** Similar panels as **Fig 9** but enforcing a minimum distance of *ε = 0*.*5 μm* between targets to reflect more realistic volume exclusion where targets are physical entities that cannot lie directly on top of each other. **B.** Similar tests for panels from **Fig 10**.
(TIF)

S1 Table Randomness test for BPs and TPs of real dendrites. The null hypothesis is uniform Poisson and we test three different alternative hypotheses:
1) *R* ≠ 1 corresponds to a clustered or regular point pattern.
2) *R* < 1 corresponds to a clustered point pattern.
3) *R* > 1 corresponds to a regular point pattern.
The table shows the percentage of cells of each type (for 2D and 3D cells and for BPs and TPs) for which the null hypothesis is rejected (i.e., p-value < 0.05) for each one of the alternative hypotheses (columns 2, 3 and 4, respectively). The p-values are computed using the Monte Carlo simulations of Poisson point cloud instances for each cell.
(DOCX)
