# Supplementary figures and images for: A regularity index for dendrites - local statistics of a neuron's input space
Source: PLoS Comput Biol. 2018 Nov 12;14(11):e1006593. doi: 10.1371/journal.pcbi.1006593 (PMC6258381; doi:10.1371/journal.pcbi.1006593)

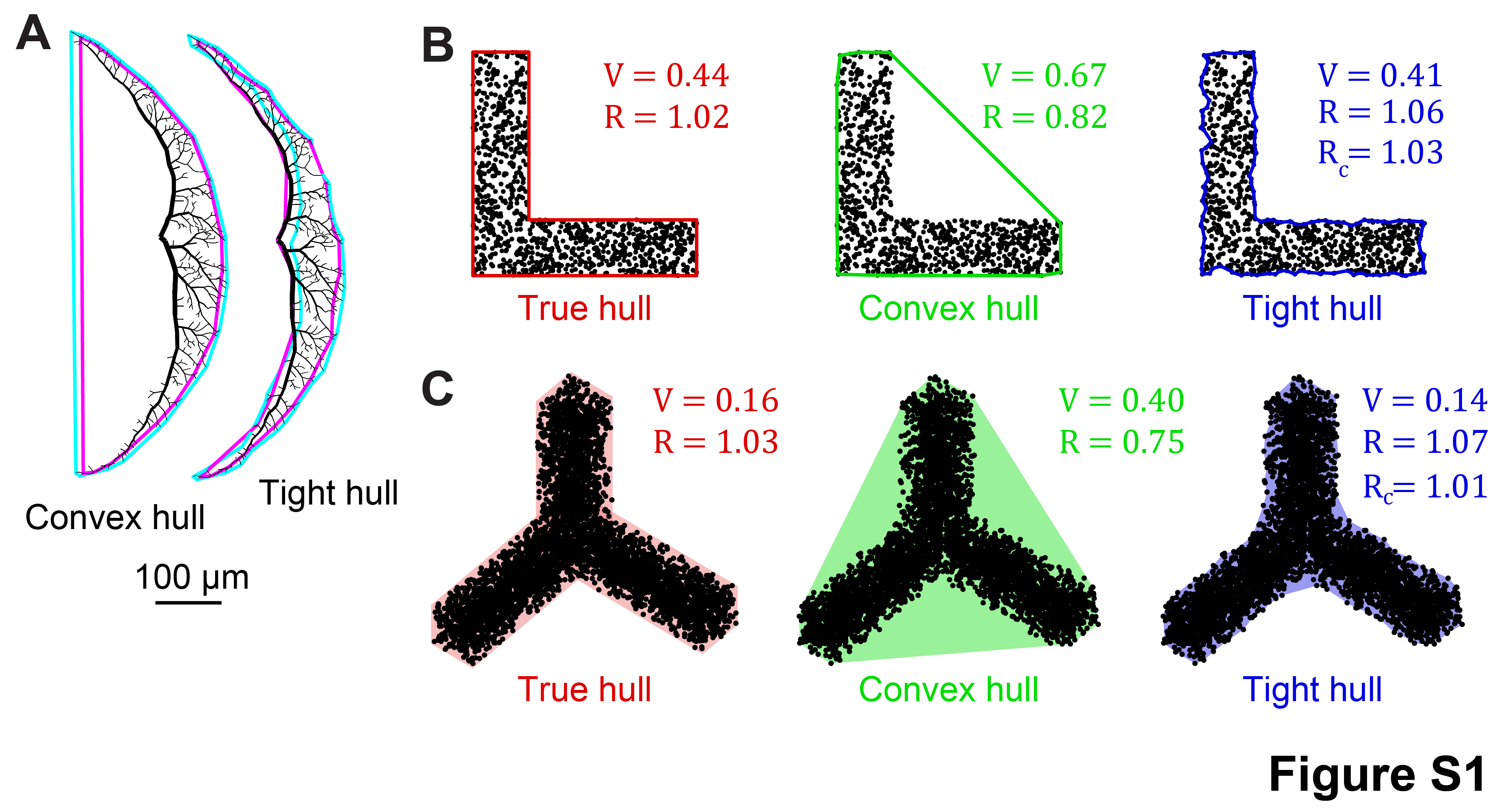

Supplement: S1 Fig — A. Example of a non-convex TC dendrite. Magenta shows the hull around BPs and cyan shows the hull around TPs. (Left) Convex hull. (Right) Tight hull. B, C. Examples of the implemented approximation to compute the R measure through a tight hull in 2D for an L-shape with 1,000 points (B), and in 3D for a double L-shape with 3,000 points (C). Expected R and volume V using the correct volume (computed analytically, red), using the convex hull (green) and using the tight hull (blue). Rc is the calculated value using our Monte Carlo (MC) approach within the tight hull to remove the remaining bias. (TIF) [file pcbi.1006593.s001.tif]

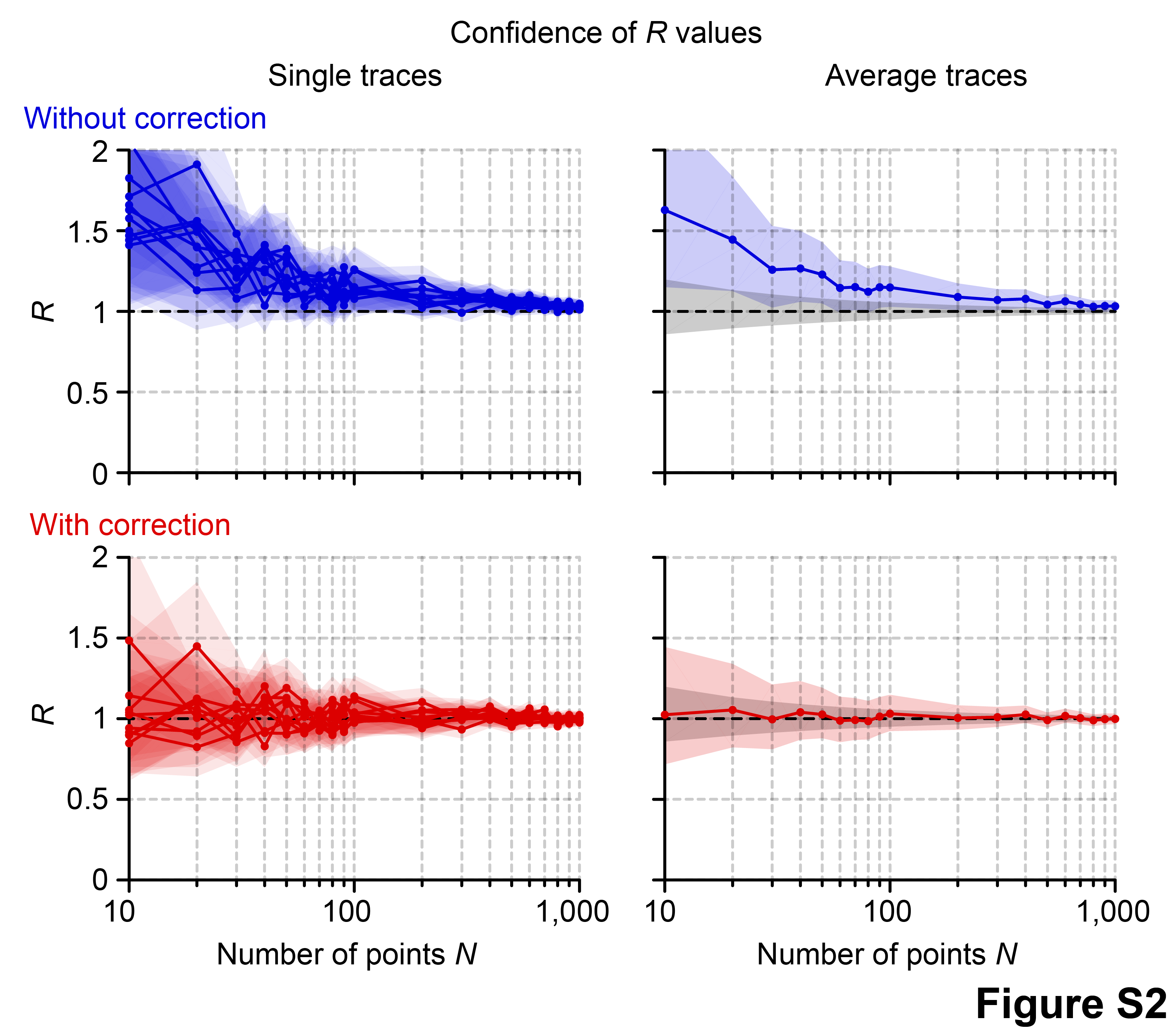

Supplement: S2 Fig — Single traces (left) and averages of 10 traces (right) for estimates of R without (top, blue straight lines) and with volume correction (bottom, red straight lines). Dots in all cases indicate the individual simulation results and shaded areas show the confidence intervals in each case. The grey shaded areas show the best possible confidence intervals calculated analytically. Each estimate of R was done with our Monte Carlo based estimator using 100 iterations. (TIF) [file pcbi.1006593.s002.tif]

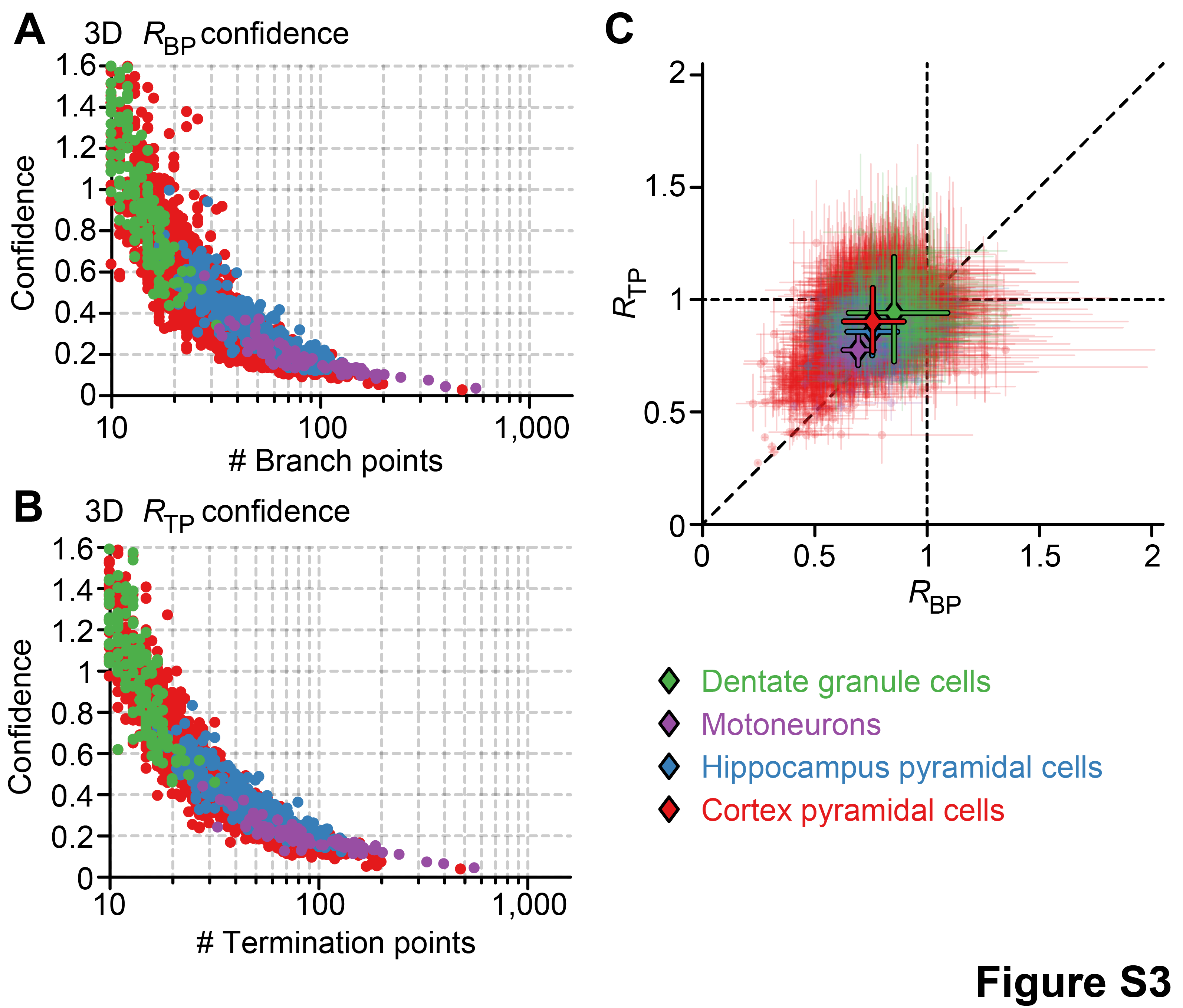

Supplement: S3 Fig — A. Confidence interval length for RBP with respect to number of BPs for 3D cells. Confidence intervals decreased with number of BPs. B. Similar graph for the confidence in the RTP measure. C. Estimated R values for BPs and TPs of 3D cells with confidence intervals. Horizontal axis shows estimated R value for sets of BPs, vertical axis estimated R value for sets of TPs of each cell. Each dot represents one cell, color coded by cell type. Horizontal and vertical whiskers indicate 95% confidence intervals for RBP and RTP, respectively. Diamonds show average values and confidence intervals for each population. (TIF) [file pcbi.1006593.s003.tif]

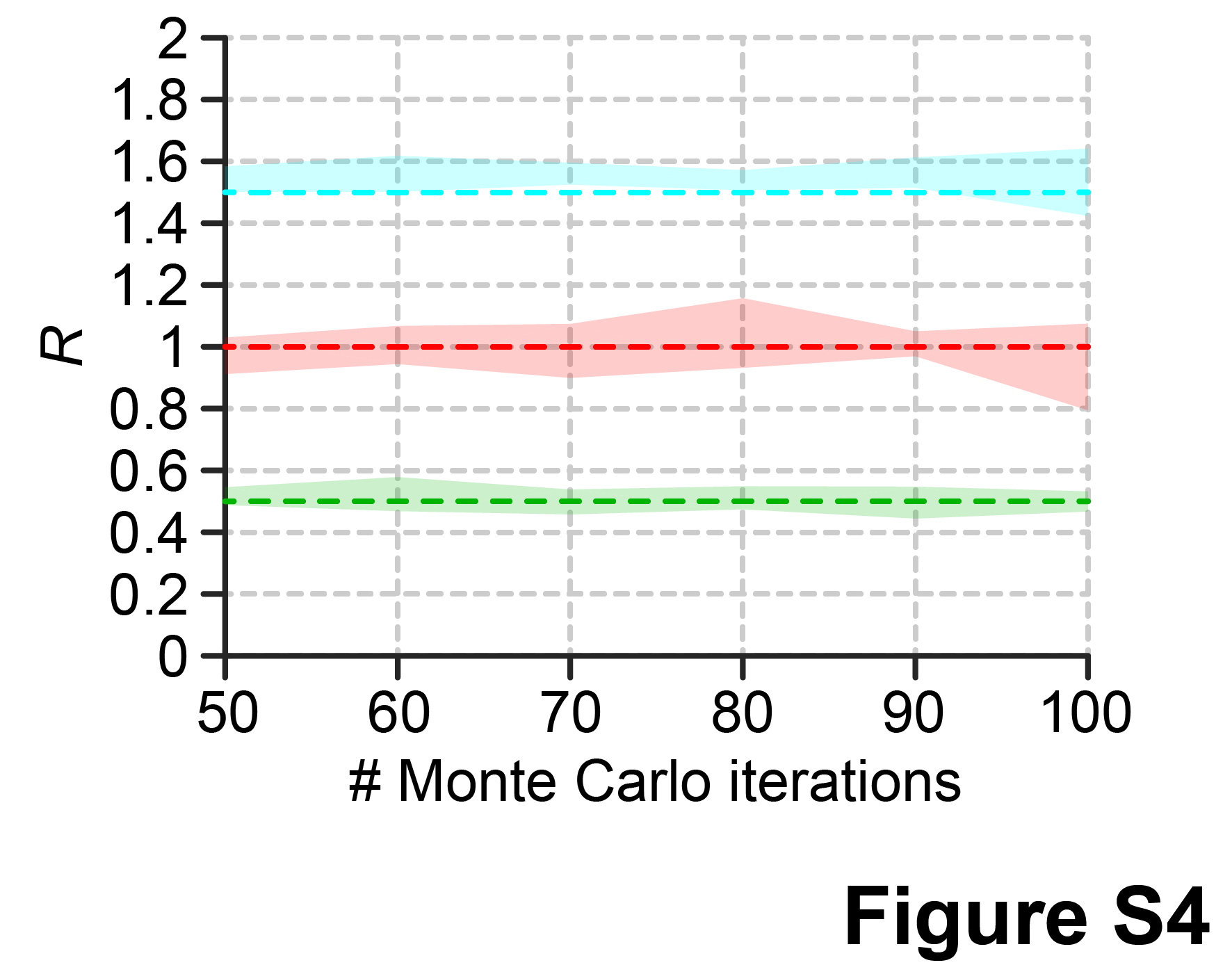

Supplement: S4 Fig — Estimated R values via MC for point clouds with known R in a square area with N = 50 points. Dashed lines show the true R values. The mean and standard deviation of 10 estimated R values are shown in green (R = 0.5), red (R = 1) and cyan (R = 1.5). Here we used from 50 to 100 Monte Carlo iterations to obtain each estimated R. (TIF) [file pcbi.1006593.s004.tif]

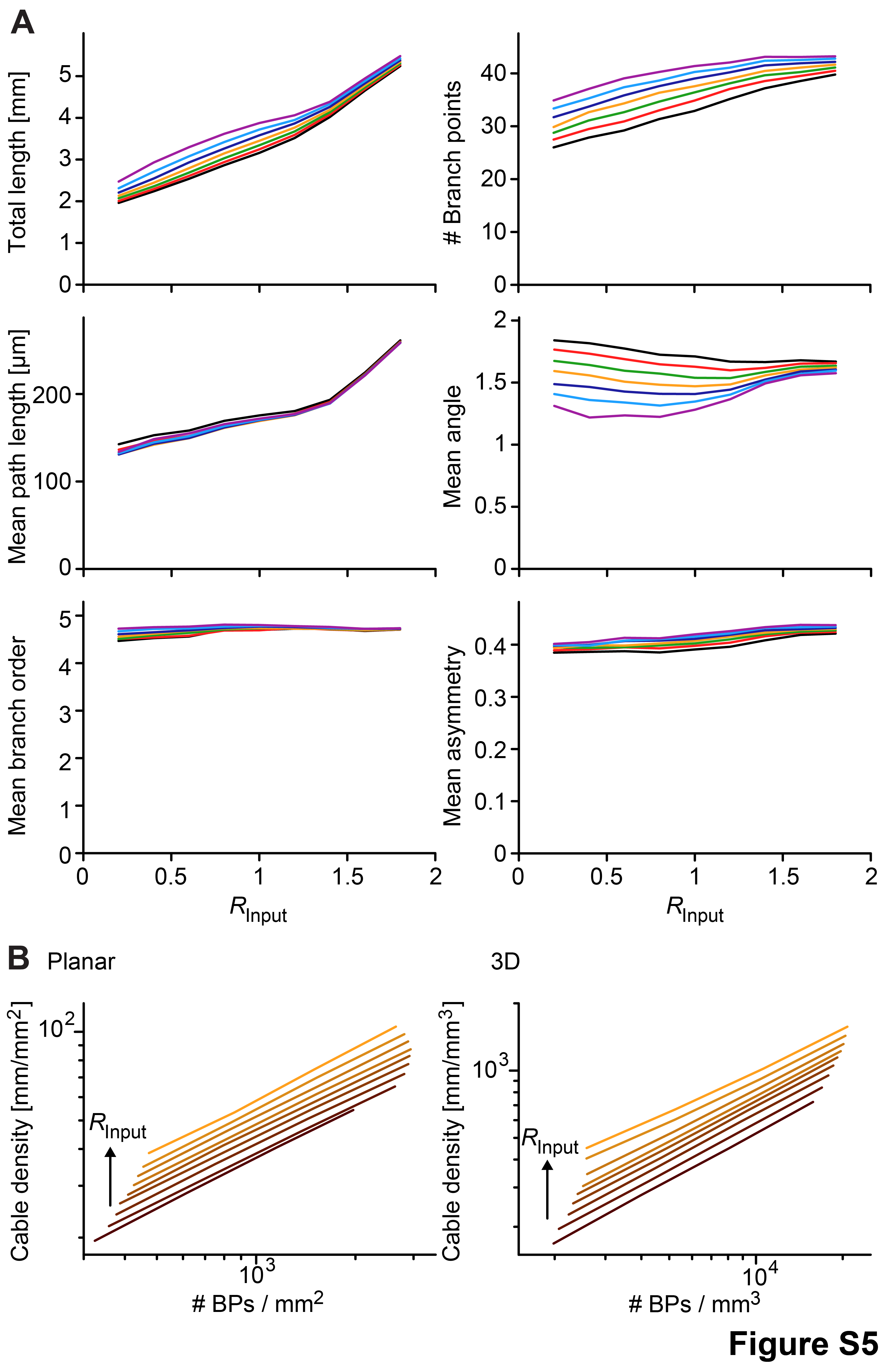

Supplement: S5 Fig — A. Similar panels as Fig 9 but enforcing a minimum distance of ε = 0.5 μm between targets to reflect more realistic volume exclusion where targets are physical entities that cannot lie directly on top of each other. B. Similar tests for panels from Fig 10. (TIF) [file pcbi.1006593.s005.tif]
