# Supplementary material for: A regularity index for dendrites - local statistics of a neuron's input space
Source: PLoS Comput Biol. 2018 Nov 12;14(11):e1006593. doi: 10.1371/journal.pcbi.1006593 (PMC6258381; doi:10.1371/journal.pcbi.1006593)
Supplement: S1 Table — The null hypothesis is uniform Poisson and we test three different alternative hypotheses: 1) R ≠ 1 corresponds to a clustered or regular point pattern. 2) R < 1 corresponds to a clustered point pattern. 3) R > 1 corresponds to a regular point pattern. The table shows the percentage of cells of each type (for 2D and 3D cells and for BPs and TPs) for which the null hypothesis is rejected (i.e., p-value < 0.05) for each one of the alternative hypotheses (columns 2, 3 and 4, respectively). The p-values are computed using the Monte Carlo simulations of Poisson point cloud instances for each cell. (DOCX) [file pcbi.1006593.s006.docx]

| **2D BPs** | **Not Random** | **Clustered** | **Regular** |
| --- | --- | --- | --- |
| Retinal ganglion cells | 22.67% | 31.99% | 1.24% |
| Purkinje cells | 13.33% | 13.33% | 6.67% |
| Fly dendritic arborization (da) neurons | 94.12% | 97.06% | 0.00% |
| Fly lobula plate tangencial cells | 58.18% | 70.91% | 0.00% |
| **2D TPs** | **Not Random** | **Clustered** | **Regular** |
| Retinal ganglion cells | 15.22% | 15.53% | 11.80% |
| Purkinje cells | 26.67% | 13.33% | 26.67% |
| Fly dendritic arborization (da) neurons | 77.94% | 79.41% | 1.47% |
| Fly lobula plate tangencial cells | 3.64% | 10.91% | 3.64% |
| **3D BPs** | **Not Random** | **Clustered** | **Regular** |
| Cortex pyramidal cells | 65.35% | 76.27% | 0.11% |
| Hippocampus pyramidal cells | 75.19% | 83.96% | 0.00% |
| Dendate granule cells | 23.38% | 34.42% | 0.00% |
| Motoneurons | 95.12% | 96.34% | 0.00% |
| **3D TPs** | **Not Random** | **Clustered** | **Regular** |
| Cortex pyramidal cells | 25.53% | 33.59% | 0.69% |
| Hippocampus pyramidal cells | 48.62% | 65.16% | 0.00% |
| Dendate granule cells | 5.19% | 9.09% | 1.95% |
| Motoneurons | 81.71% | 90.24% | 0.00% |
